# Ginsenoside Rh1 Modulates GABAergic Inhibitory Homeostasis and Mitochondrial Quality Control During Lead (Pb)-Associated Neuronal Dysfunction

**DOI:** 10.3390/molecules31162801

**Published:** 2026-08-11

**Authors:** Xiang Li, Tingting Wang, Linfeng Mo, Huixin Cao, Zhongting Lv, Jia Yu, Shuang Liu, Yantong Sun, Tianli Chen

**Affiliations:** 1 Department of Pharmacology, College of Basic Medical Sciences, The Medical Basic Research Innovation Center of Airway Disease in North China, Key Laboratory of Pathobiology, Ministry of Education, Jilin University, Changchun 130021, China; lixiang001@jlu.edu.cn (X.L.); molf9924@mails.jlu.edu.cn (L.M.); tianhx9924@mails.jlu.edu.cn (H.C.); 2 Ginseng Research Institute, Jilin University, Changchun 130021, China; 3 College of Food Science and Engineering, Jilin University, Changchun 130062, China; wangtt9924@mails.jlu.edu.cn (T.W.); ztlv24@mails.jlu.edu.cn (Z.L.); yujia25@mails.jlu.edu.cn (J.Y.); shuangliu25@mails.jlu.edu.cn (S.L.); 4 School of Pharmaceutical Sciences, Jilin University, Changchun 130021, China; 5 School of Pharmaceutical Sciences, Changchun University of Chinese Medicine, Changchun 130000, China

**Keywords:** ginsenoside Rh1, Pb metal neurotoxicity, GABAergic homeostasis, mitophagy

## Abstract

Disruption of neuronal excitation–inhibition balance and mitochondrial quality control may contribute importantly to lead (Pb)-induced neurotoxicity, but nutritional modulators targeting these processes remain poorly characterized. This study investigated the protective effects and underlying mechanisms of ginsenoside Rh1, a ginseng-derived bioactive compound, in Pb-exposed mice and Pb-treated HT22 hippocampal cells. Chronic Pb exposure caused spatial recognition deficits, reduced exploratory activity, anxiety-like behaviors, and marked neuronal injury, accompanied by Pb accumulation in blood and brain tissues, elevated IL-1β, TNF-α, and IL-6 levels, oxidative stress, reduced Gama-aminobutyric acid (GABA) content, and dysregulated NKCC1/KCC2 expression. In HT22 cells, Pb increased intracellular ROS generation and disrupted mitophagy-related signaling, as indicated by alterations in PINK1, Parkin, LC3, P62, and GABARAP. Rh1 treatment alleviated Pb-induced behavioral abnormalities and neuronal pathology, reduced Pb burden, suppressed neuroinflammatory responses, enhanced antioxidant defenses, improved Pb-associated alterations in GABAergic regulation, and modulated mitochondrial quality-control signaling in vivo and in vitro. These findings suggest that Rh1 may represent a promising nutritional intervention strategy for mitigating Pb-associated neurotoxicity.

## 1. Introduction

Neuronal function depends on the coordinated maintenance of inhibitory neurotransmission and mitochondrial quality control [1]. Disruption of either process can impair hippocampal activity, alter behavioral performance, and increase neuronal vulnerability to toxic insults [2,3]. In the context of chronic lead (Pb) exposure, increasing evidence suggests that neurotoxicity is not limited to generalized oxidative injury but may also involve disturbance of excitation-inhibition balance, abnormal chloride transporter regulation, and alterations in mitochondrial quality-control processes [4,5]. However, the extent to which Pb accumulation is accompanied by coordinated alterations in GABA-associated regulation and mitophagy-related signaling remains insufficiently characterized.

Pb is a cumulative heavy metal contaminant that can enter the human body through contaminated food, drinking water, soil, and environmental media [6,7,8]. After absorption, Pb can persist in blood and target organs and is closely associated with neurodevelopmental impairment, cognitive decline, emotional abnormalities, and neuronal injury [9,10]. Although exposure control and chelation therapy remain important strategies for managing Pb poisoning, they have limited applicability for mitigating chronic Pb-associated neuronal injury, particularly under long-term dietary or environmental exposure conditions [11]. Therefore, identifying ginseng-derived bioactive compounds that can attenuate Pb-induced neurotoxicity and clarifying their underlying mechanisms may provide insights into potential preventive approaches for Pb-associated neuronal injury.

Among the neural systems affected by Pb, the GABAergic inhibitory system is of particular importance [12]. The inhibitory efficacy of GABA_A_ receptor-mediated signaling largely depends on the transmembrane chloride gradient, which is maintained by the coordinated activities of the sodium–potassium–chloride cotransporter 1 (NKCC1) and potassium–chloride cotransporter 2 (KCC2) [13]. Under physiological conditions, KCC2-mediated chloride extrusion supports hyperpolarizing GABAergic inhibition, whereas excessive NKCC1 activity or reduced KCC2 expression may weaken inhibitory control and shift GABAergic responses toward a less inhibitory or even depolarizing state [14]. Therefore, alterations in NKCC1/KCC2 balance may represent one molecular feature associated with Pb-induced disturbances in neuronal inhibition and behavioral abnormalities. In addition, because GABA metabolism and synaptic inhibition are closely associated with mitochondrial energy homeostasis, Pb-induced mitochondrial dysfunction may further aggravate GABAergic dysregulation [15].

Mitochondrial injury may further contribute to this inhibitory imbalance [16]. Pb exposure can promote mitochondrial oxidative damage, excessive reactive oxygen species (ROS) generation, loss of mitochondrial membrane potential, and impairment of mitochondrial quality-control processes [10]. In parallel, Pb-induced inflammatory responses may further amplify neuronal damage by increasing the release of pro-inflammatory cytokines and aggravating redox imbalance. The depletion of endogenous antioxidants, such as glutathione (GSH) and superoxide dismutase (SOD), together with increased lipid peroxidation, is considered an important biochemical feature of Pb-induced oxidative injury [17,18]. Mitophagy, a selective form of autophagy responsible for the removal of damaged mitochondria, is mainly regulated by the PINK1/Parkin pathway and is essential for maintaining mitochondrial integrity in neurons [19,20]. However, either excessive activation or insufficient mitophagic clearance may contribute to neuronal dysfunction rather than protection. Emerging evidence indicates that Pb exposure perturbs mitophagy-related signaling, suggesting that alterations in mitochondrial quality-control signaling may be involved in Pb-induced neurotoxicity [10]. Nevertheless, whether alterations in mitophagy-related signaling are associated with changes in GABAergic regulation during Pb-induced neuronal injury remains insufficiently understood.

**Figure 1:**
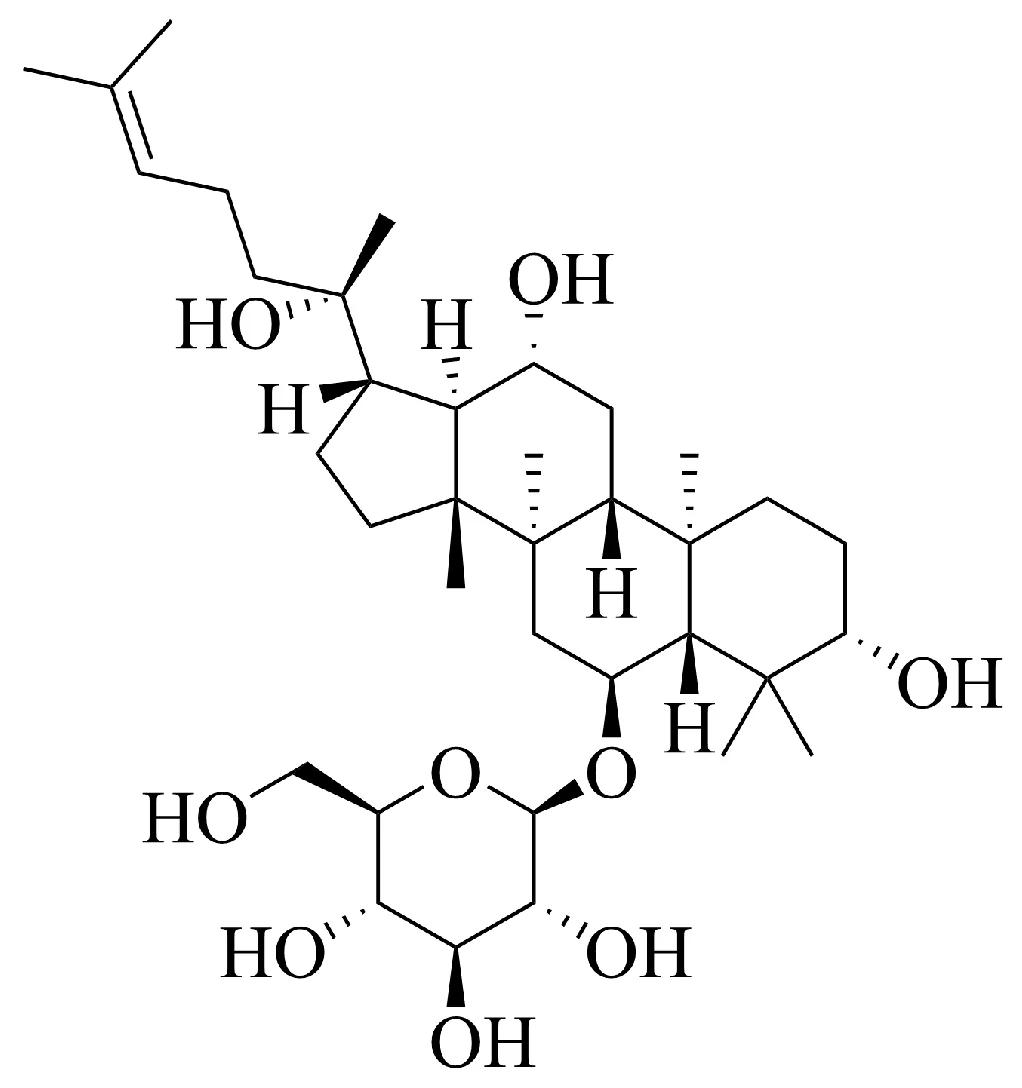
Chemical structure of ginsenoside Rh1.

Ginsenoside Rh1 is a protopanaxatriol-type saponin derived from *Panax ginseng*, a medicinal plant widely used in traditional medicine and as an herbal supplement. As a ginseng-derived bioactive compound, Rh1 has attracted attention because of its antioxidant, anti-inflammatory, anti-apoptotic, and neuroprotective activities in several experimental models [21,22]. Compared with crude herbal extracts, Rh1 is structurally well characterized (Figure 1), making it suitable for investigating the biological functions of a specific ginseng-derived bioactive compound in toxicological contexts. Previous studies have suggested that Rh1 can attenuate oxidative stress and mitochondrial injury [23,24,25]. However, its protective effects against Pb-induced neurotoxicity, especially under chronic exposure conditions relevant to food safety and environmental health, have not been fully elucidated. In particular, it remains unclear whether Rh1 can influence Pb burden, suppress inflammatory activation, antioxidant responses, NKCC1/KCC2-associated regulation, and mitophagy-related signaling in Pb-induced neuronal injury.

In the present study, chronic Pb-exposed mice and Pb-treated HT22 cells were used to evaluate the protective effects of Rh1 and to explore the underlying mechanisms. Behavioral performance, brain histopathology, blood and brain Pb levels, serum inflammatory cytokines, brain oxidative stress biomarkers, GABA content, NKCC1/KCC2 expression, ROS production, and mitophagy-related markers were systematically examined. This study aimed to evaluate whether the ginseng-derived bioactive compound Rh1 attenuates Pb-induced neuronal injury and to investigate its association with Pb burden, inflammatory and oxidative stress responses, GABA-associated regulation, and mitochondrial quality-control signaling. These findings provide preliminary mechanistic evidence supporting further investigation of Rh1 as a ginseng-derived bioactive compound with potential relevance to Pb-associated neuronal injury.

## 2. Results

### 2.1. Rh1 Supplementation Ameliorated Pb-Induced Neurobehavioral Abnormalities in Mice

**Figure 2:**
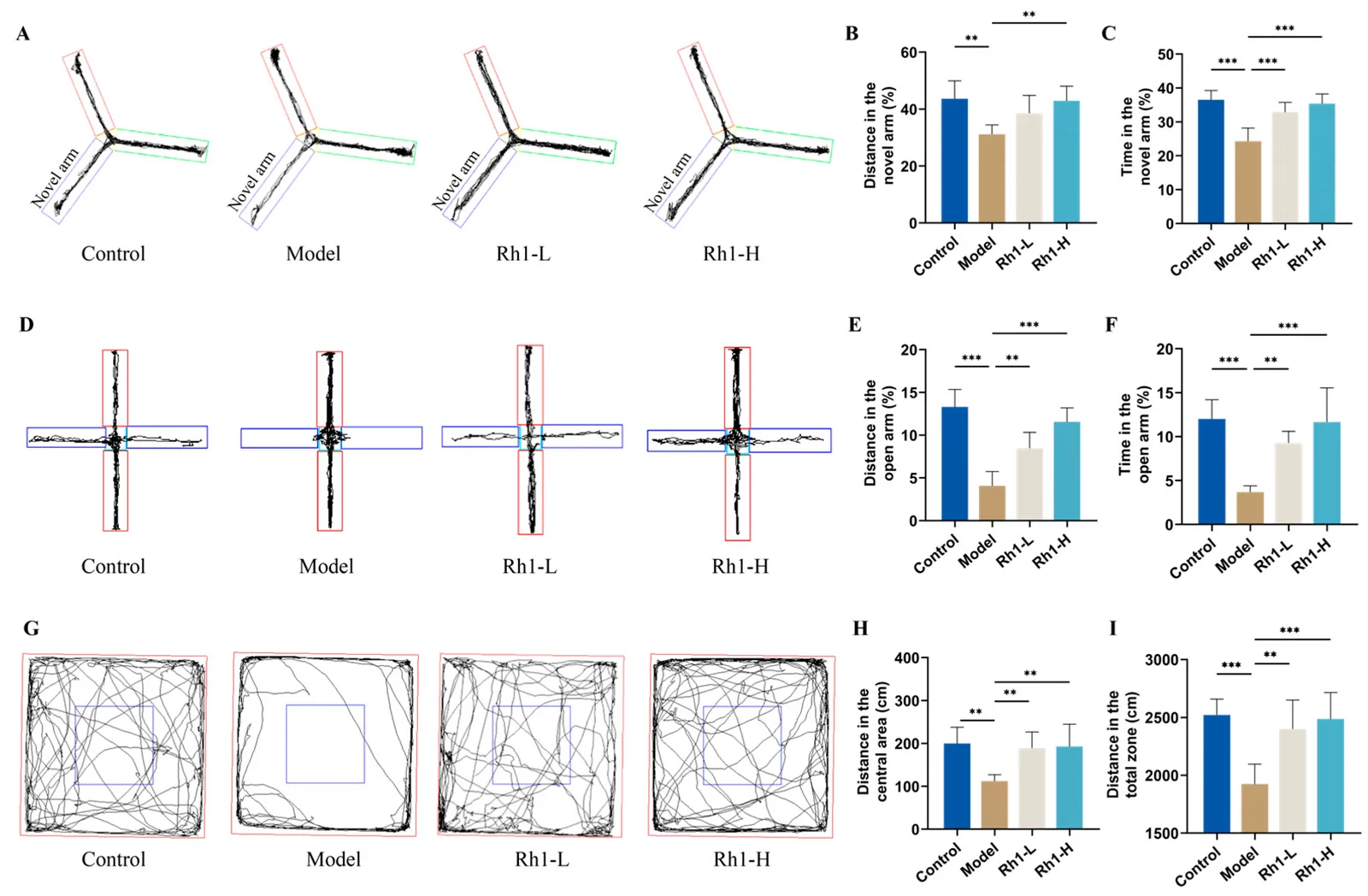
Rh1 supplementation attenuated Pb-associated neurobehavioral abnormalities in mice: (**A**) Representative movement trajectories in the Y-maze test. (**B**,**C**) Quantitative analysis of novel arm entries and distance traveled in the novel arm. (**D**) Representative movement trajectories in the elevated plus maze. (**E**,**F**) Quantitative analysis of open-arm distance and percentage of time spent in the open arms. (**G**) Representative movement trajectories in the open-field test. (**H**,**I**) Quantitative analysis of time spent in the central zone and total distance traveled. Data are presented as the mean ± SD (*n* = 8, ** *p* < 0.01, *** *p* < 0.001). Control, untreated control group; Model, Pb-exposed model group; Rh1-L, Pb-exposed group treated with low-dose ginsenoside Rh1; Rh1-H, Pb-exposed group treated with high-dose ginsenoside Rh1; SD, standard deviation. In the trajectory figures, specific areas are indicated by colored borders: in the Y-maze (**A**), the blue border indicates the novel arm; in the elevated plus maze (**D**), the blue borders indicate the open arms and the red borders indicate the closed arms; in the open-field test (**G**), the blue square indicates the central zone.

Behavioral tests were performed to evaluate the effects of the ginseng-derived bioactive compound Rh1 on Pb-induced neurobehavioral dysfunction in mice. Mice were exposed to drinking water containing 100 ppm lead acetate for 10 weeks, and Rh1 was administered during weeks 7–10 of Pb exposure. In the Y-maze novel-arm recognition test, Pb-exposed mice showed significantly fewer entries into the novel arm and a shorter distance traveled in the novel arm than control mice, indicating impaired exploratory activity and spatial recognition memory. Representative movement trajectories also showed reduced exploration of the novel arm in the Pb model group. Rh1 supplementation increased the number of novel-arm entries and the distance traveled in the novel arm, with the high-dose Rh1 group showing a more pronounced effect (Figure 2A–C).

In the elevated plus maze, chronic Pb exposure significantly reduced open-arm exploration, as reflected by decreases in both the distance traveled in the open arms and the percentage of time spent in the open arms, consistent with increased anxiety-like behavior. Rh1 supplementation increased open-arm activity compared with the Pb model group, indicating an alleviation of Pb-induced anxiety-like responses (Figure 2D–F).

Consistent with these observations, Pb-exposed mice exhibited reduced central-zone exploration and locomotor activity in the open-field test, as reflected by a shorter duration in the central zone and a decreased total distance traveled. Representative trajectories showed that Pb-exposed mice predominantly moved along the peripheral area, whereas control mice explored both the central and peripheral regions. Rh1 supplementation increased central-zone exploration and total distance traveled compared with the Pb model group (Figure 2G–I).

Collectively, these results show that chronic Pb exposure was associated with impairments in spatial recognition, exploratory behavior, and increased anxiety-like behavior, whereas Rh1 supplementation attenuated these Pb-associated neurobehavioral abnormalities.

### 2.2. Rh1 Supplementation Alleviated Pb-Associated Histopathological Alterations in Brain Tissue

**Figure 3:**
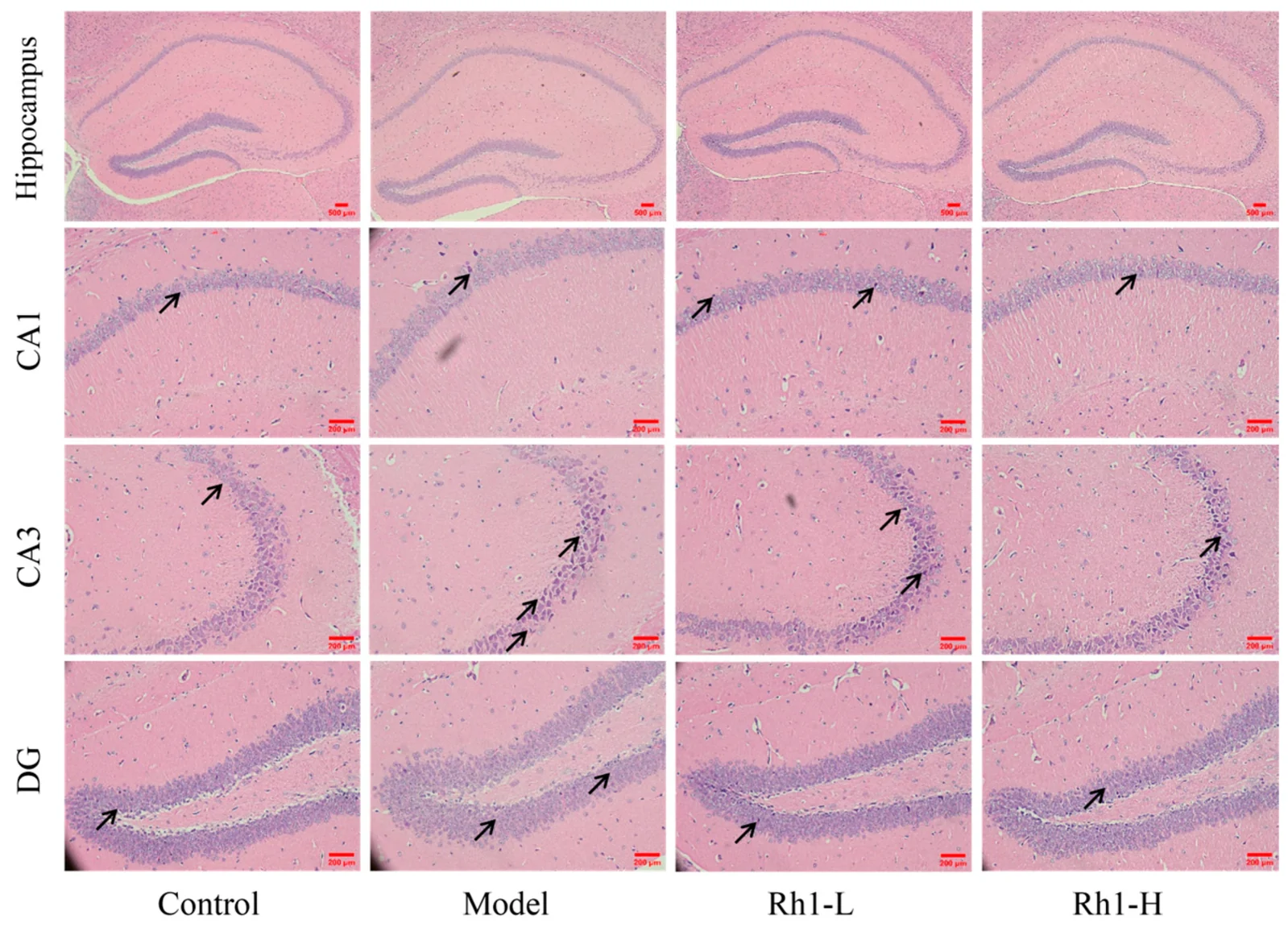
Rh1 supplementation alleviated Pb-associated histopathological alterations in mouse hippocampal tissue. Representative H&E-stained sections showing the overall hippocampus and the CA1, CA3, and DG regions in the Control, Model, Rh1-L, and Rh1-H groups. Black arrows indicate representative neurons and morphological features in the indicated regions. Scale bars are 500 μm in the whole-hippocampus images and 200 μm in the CA1, CA3, and DG images. Control, untreated control group; Model, Pb-exposed model group; Rh1-L, Pb-exposed group treated with low-dose ginsenoside Rh1; Rh1-H, Pb-exposed group treated with high-dose ginsenoside Rh1; CA1 and CA3, cornu ammonis regions 1 and 3; DG, dentate gyrus.

Hematoxylin and eosin (H&E) staining was performed to evaluate the effects of Rh1 on Pb-associated structural alterations in brain tissue. As shown in Figure 3, the control group exhibited well-preserved brain histoarchitecture, with regularly arranged neuronal layers, dense cellular distribution, clear nuclear morphology, and uniform cytoplasmic staining. In contrast, Pb-exposed mice showed evident histopathological abnormalities, including disorganized neuronal arrangement, apparently reduced cellular density, nuclear pyknosis, shrunken cell bodies, and enlarged intercellular spaces, findings consistent with neuronal injury.

Rh1 supplementation alleviated these histopathological alterations. In the Rh1-L group, neuronal disorganization and morphological abnormalities appeared less evident than those in the Pb model group, although mild structural abnormalities remained visible. The Rh1-H group exhibited better preservation of tissue morphology, including more regularly arranged neuronal layers and fewer visibly pyknotic or shrunken cells compared with the Pb model group. These findings suggest that Rh1 supplementation attenuated Pb-associated histopathological alterations in brain tissue.

### 2.3. Rh1 Supplementation Reduced Pb Accumulation, Systemic Inflammation, and Brain Oxidative Stress in Pb-Exposed Mice

**Figure 4:**
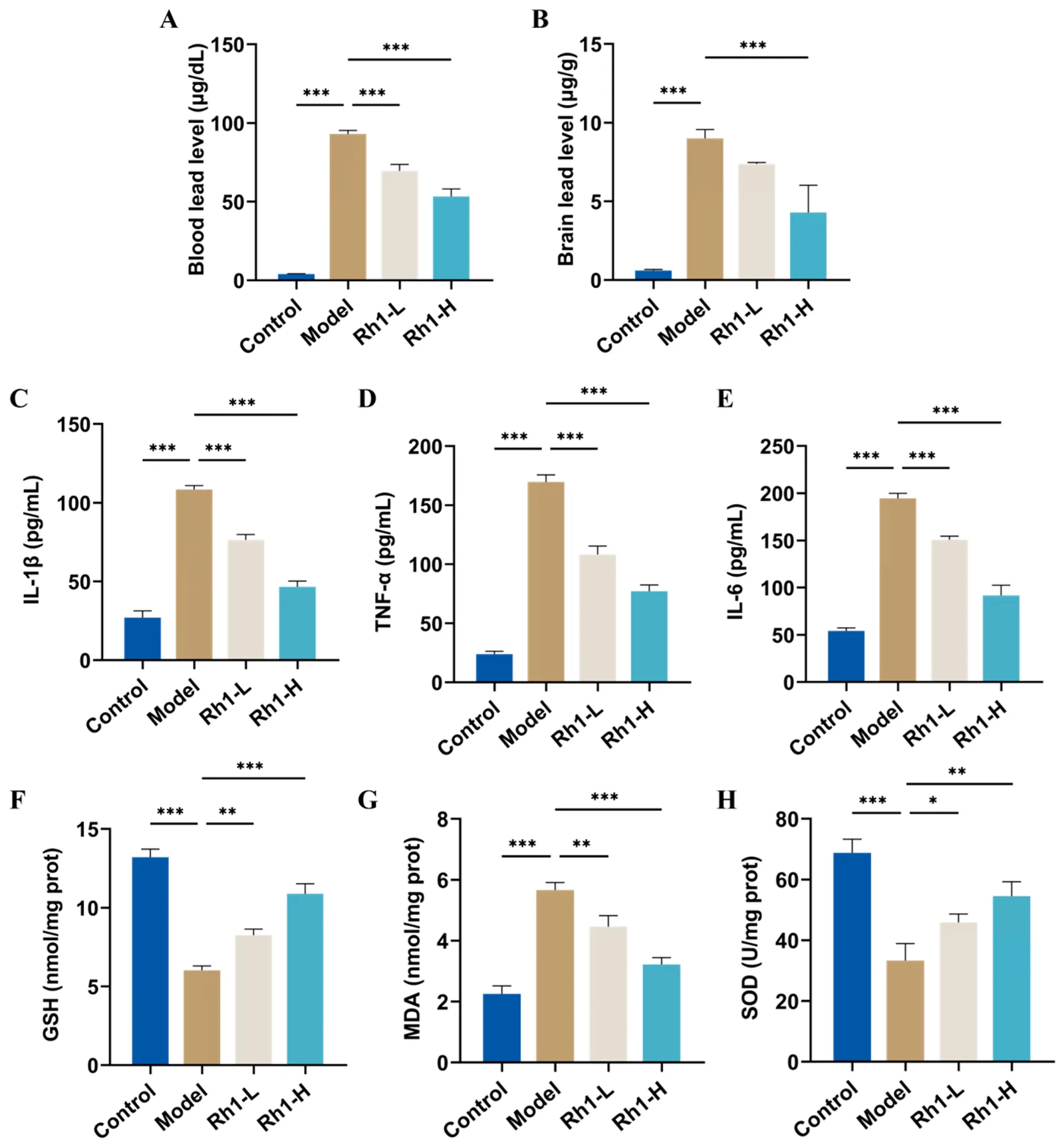
Rh1 supplementation attenuated Pb-associated alterations in Pb burden, systemic inflammation, and brain oxidative stress-related indicators in Pb-exposed mice: (**A**) Blood Pb levels. (**B**) Brain Pb levels. (**C**–**E**) Serum levels of pro-inflammatory cytokines IL-1β, TNF-α, and IL-6. (**F**–**H**) Oxidative stress-related indicators in brain tissue, including GSH content, MDA level, and SOD activity. Data are presented as the mean ± SD (*n* = 3, * *p* < 0.05, ** *p* < 0.01, *** *p* < 0.001). Control, untreated control group; Model, Pb-exposed model group; Rh1-L, Pb-exposed group treated with low-dose ginsenoside Rh1; Rh1-H, Pb-exposed group treated with high-dose ginsenoside Rh1.

Pb accumulation, systemic inflammation, and oxidative stress were assessed to determine whether Rh1 influenced key toxicological responses associated with chronic Pb exposure. As shown in Figure 4, Pb exposure resulted in increased Pb burden, elevated pro-inflammatory cytokine levels, and alterations in antioxidant-related indicators, whereas Rh1 supplementation was associated with attenuation of these changes.

Compared with the control group, mice in the Pb model group showed significantly increased Pb levels in both blood and brain tissue, confirming the successful establishment of the chronic Pb exposure model (Figure 4A,B). Rh1 supplementation significantly reduced blood Pb concentrations in both the low- and high-dose groups. For brain Pb levels, a significant reduction was observed only in the high-dose Rh1 group, whereas low-dose Rh1 did not significantly reduce brain Pb accumulation compared with the Pb model group (Figure 4B). These results indicate that Rh1 supplementation was associated with reduced Pb burden, particularly in the high-dose group.

Serum pro-inflammatory cytokines were further measured to evaluate systemic inflammatory responses. Pb exposure significantly increased serum interleukin-1β (IL-1β), tumor necrosis factor-α (TNF-α), and interleukin-6 (IL-6) levels compared with the control group (Figure 4C–E), indicating inflammatory activation. Rh1 supplementation decreased the levels of these cytokines, with a more evident effect observed in the high-dose group. These results suggest that Rh1 supplementation was associated with attenuation of Pb-associated systemic inflammatory responses.

Oxidative stress in brain tissue was evaluated by measuring GSH content, malondialdehyde (MDA) levels, and SOD activity. Pb exposure significantly decreased GSH levels and SOD activity, while increasing MDA content in brain tissue (Figure 4F–H), indicating impaired antioxidant defense and enhanced lipid peroxidation. Rh1 supplementation was associated with increased GSH content and SOD activity and reduced MDA levels compared with the Pb model group. These alterations were more apparent in the high-dose Rh1 group.

### 2.4. Rh1 Supplementation Improved Pb-Associated Alterations in NKCC1/KCC2 Expression and GABA Content in Brain Tissue

**Figure 5:**
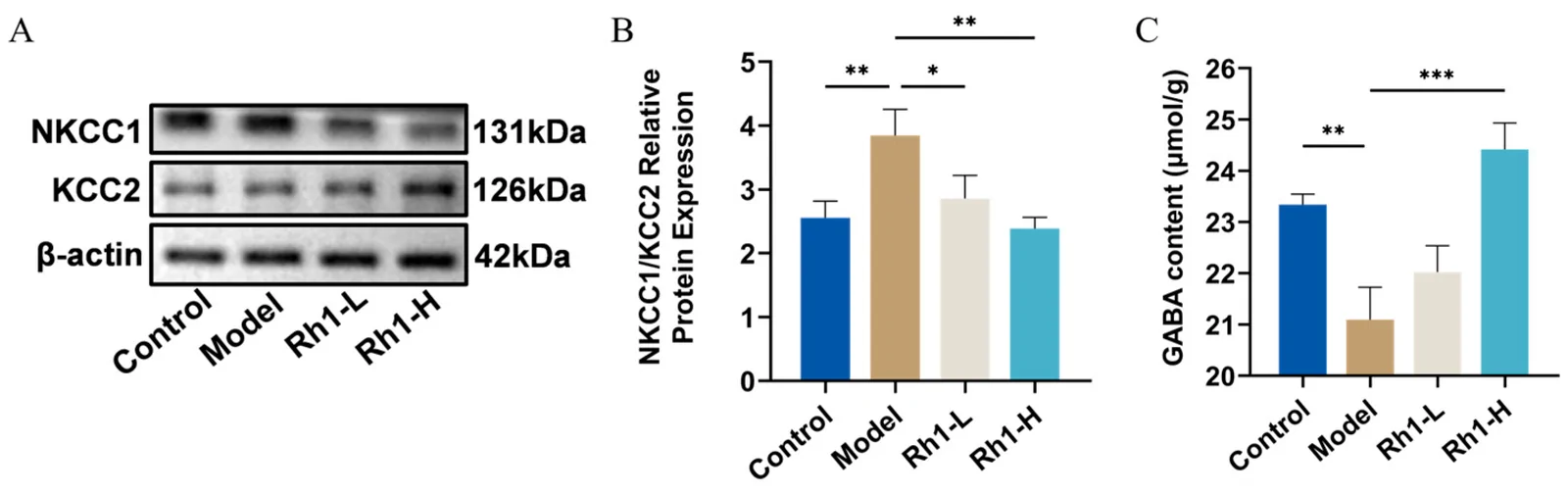
Rh1 supplementation attenuated Pb-associated alterations in NKCC1/KCC2 expression and GABA content in brain tissue: (**A**) Representative Western blot images of NKCC1 and KCC2 protein expression. (**B**) Quantitative analysis of the NKCC1/KCC2 protein expression ratio. (**C**) Quantification of GABA content in brain tissue. Data are presented as the mean ± SD (*n* = 3, * *p* < 0.05, ** *p* < 0.01, *** *p* < 0.001). Control, untreated control group; Model, Pb-exposed model group; Rh1-L, Pb-exposed group treated with low-dose ginsenoside Rh1; Rh1-H, Pb-exposed group treated with high-dose ginsenoside Rh1.

To determine whether Rh1 was associated with changes in Pb-related GABA-associated regulation, NKCC1 and KCC2 protein expressions were assessed by Western blotting. As shown in Figure 5A,B, chronic Pb exposure significantly increased the NKCC1/KCC2 ratio compared with the control group, indicating disruption of chloride transporter homeostasis. Rh1 supplementation reduced the Pb-induced elevation of the NKCC1/KCC2 ratio, with the high-dose Rh1 group showing a more pronounced effect.

Consistently, Pb exposure significantly decreased GABA content in brain tissue compared with the control group. Rh1 supplementation increased brain GABA content compared with the Pb model group, and this increase was more evident in the high-dose group (Figure 5C). These results indicate that Rh1 treatment was associated with attenuation of Pb-associated alterations in NKCC1/KCC2 expression and GABA content, suggesting a potential involvement of GABA-associated regulatory processes in the protective effects of Rh1.

### 2.5. Rh1 Supplementation Modulated Mitophagy-Related Signaling in the Brain of Pb-Exposed Mice

**Figure 6:**
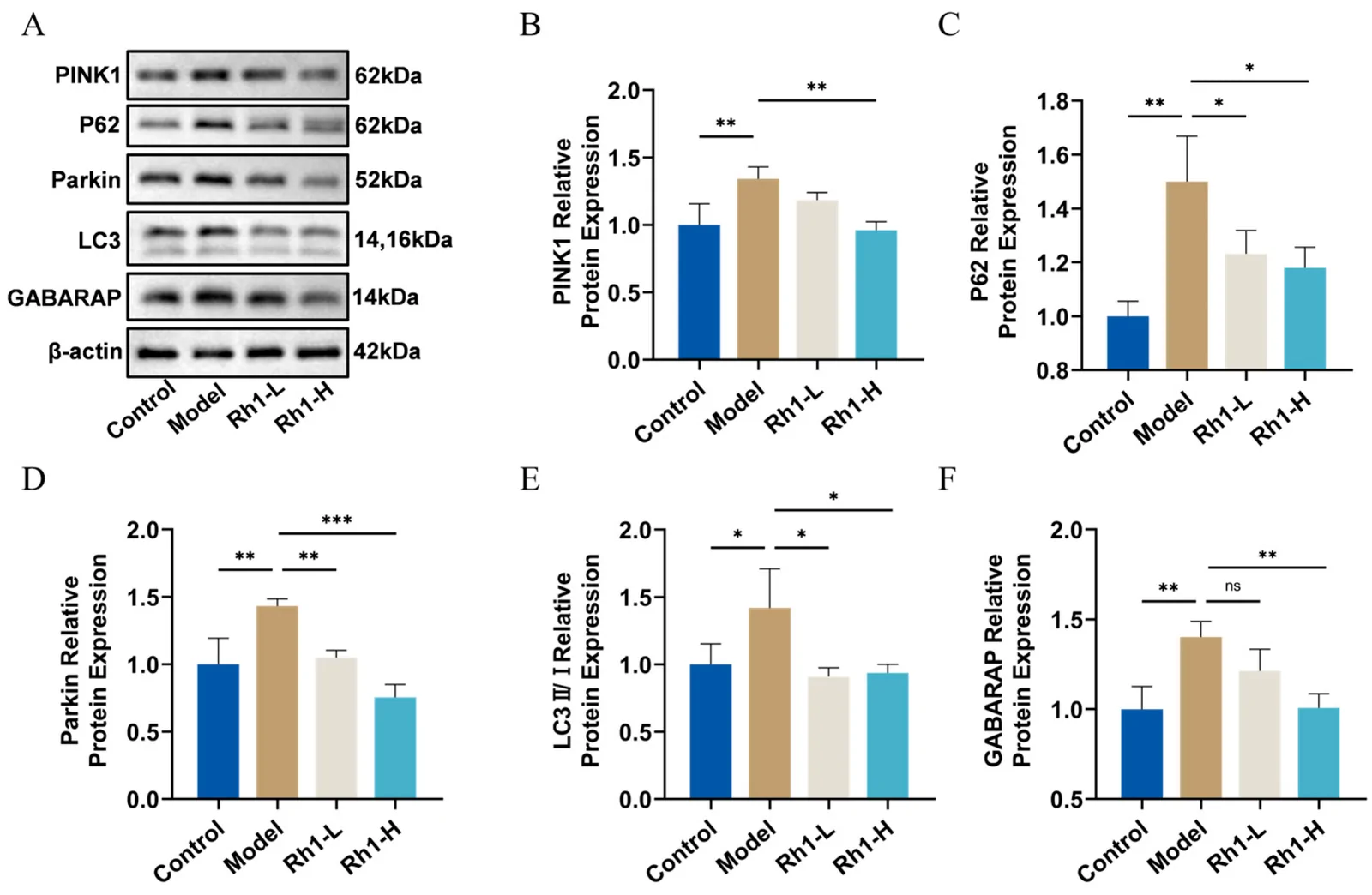
Rh1 supplementation modulated mitophagy-related protein expression in the brain of Pb-exposed mice: (**A**) Representative Western blot images of PINK1, Parkin, P62, LC3, GABARAP, and β-actin. (**B**–**F**) Quantitative analysis of PINK1, P62, Parkin, LC3-II/LC3-I ratio, and GABARAP protein expression. Data are presented as the mean ± SD (*n* = 3, * *p* < 0.05, ** *p* < 0.01, *** *p* < 0.001, ns, not significant). Control, untreated control group; Model, Pb-exposed model group; Rh1-L, Pb-exposed group treated with low-dose ginsenoside Rh1; Rh1-H, Pb-exposed group treated with high-dose ginsenoside Rh1.

The expression of mitophagy-related proteins was examined to determine whether Rh1 affected mitochondrial quality-control responses under Pb exposure. As shown in Figure 6**,** chronic Pb exposure significantly increased the protein expression levels of PINK1, Parkin, P62, and GABARAP and elevated the LC3-II/LC3-I ratio compared with the control group. These results indicate alterations in mitophagy-related and autophagy-associated protein expression under Pb exposure.

Rh1 supplementation attenuated these Pb-induced alterations. Compared with the Pb model group, Rh1 reduced the expression levels of PINK1, Parkin, P62, and GABARAP and decreased the LC3-II/LC3-I ratio. The regulatory effect was more evident in the high-dose Rh1 group. These findings suggest that Rh1 was associated with the attenuation of Pb-associated alterations in mitochondrial quality-control and mitophagy-related signaling in brain tissue.

### 2.6. Selection of Effective Rh1 Concentrations in HT22 Cells

**Figure 7:**
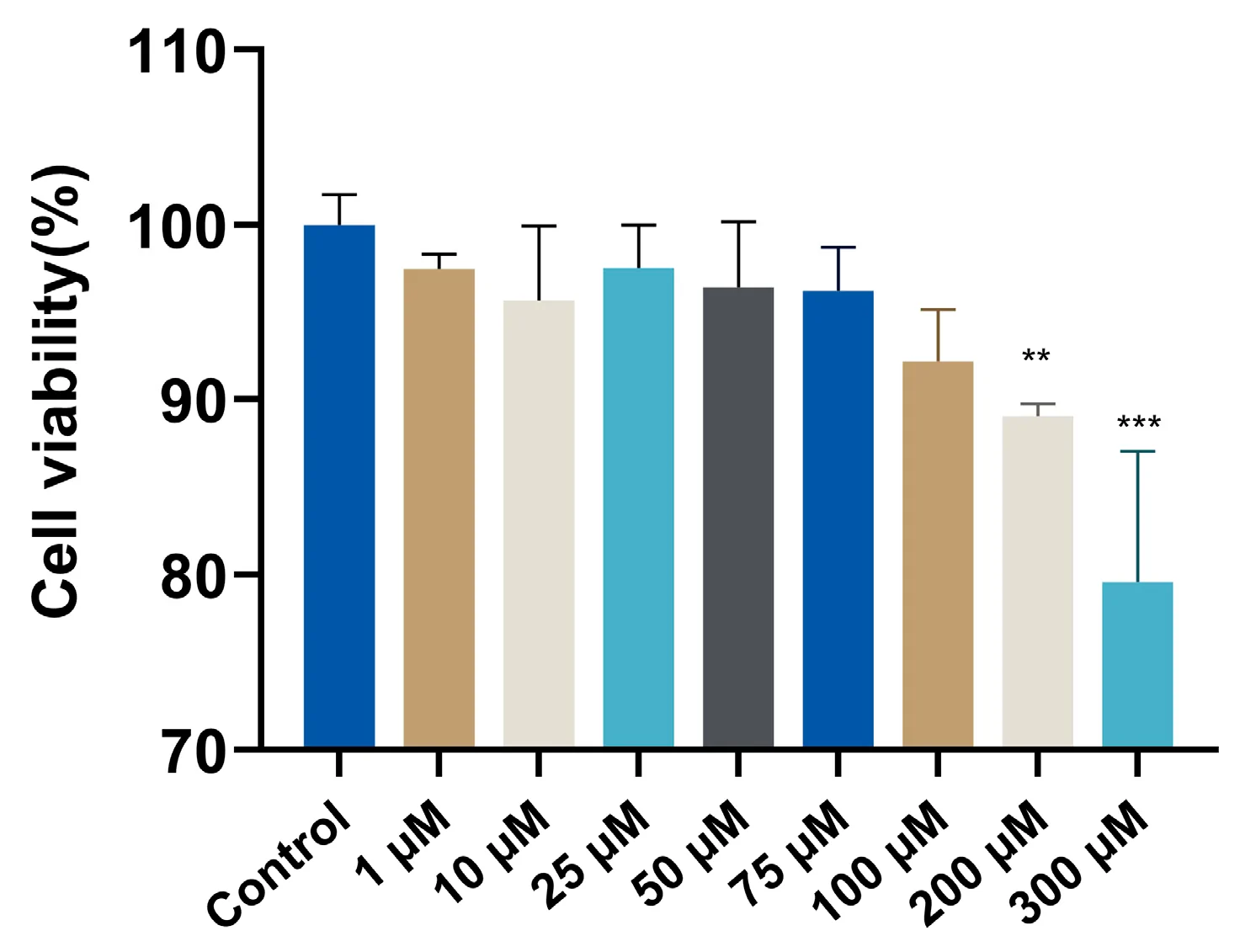
Effects of different concentrations of ginsenoside Rh1 on HT22 cell viability. HT22 cell viability was measured by CCK-8 assay after treatment with different concentrations of Rh1 for 24 h. Data are presented as the mean ± SD (*n* = 3, ** *p* < 0.01 and *** *p* < 0.001 versus the control group).

To determine appropriate Rh1 concentrations for subsequent in vitro experiments, HT22 cell viability was assessed using the Cell Counting Kit-8 (CCK-8) assay. As shown in Figure 7, Rh1 at concentrations ranging from 1 to 100 μM did not significantly affect cell viability compared with the control group, indicating no obvious cytotoxicity within this concentration range. In contrast, 200 and 300 μM Rh1 significantly reduced cell viability, indicating reduced cell viability at higher concentrations. Therefore, 10 and 100 μM Rh1 were selected as the low- and high-concentration treatments, respectively, for subsequent cellular experiments.

### 2.7. Rh1 Attenuated Pb-Associated Alterations in Autophagy- and Mitophagy-Related Markers in HT22 Cells

**Figure 8:**
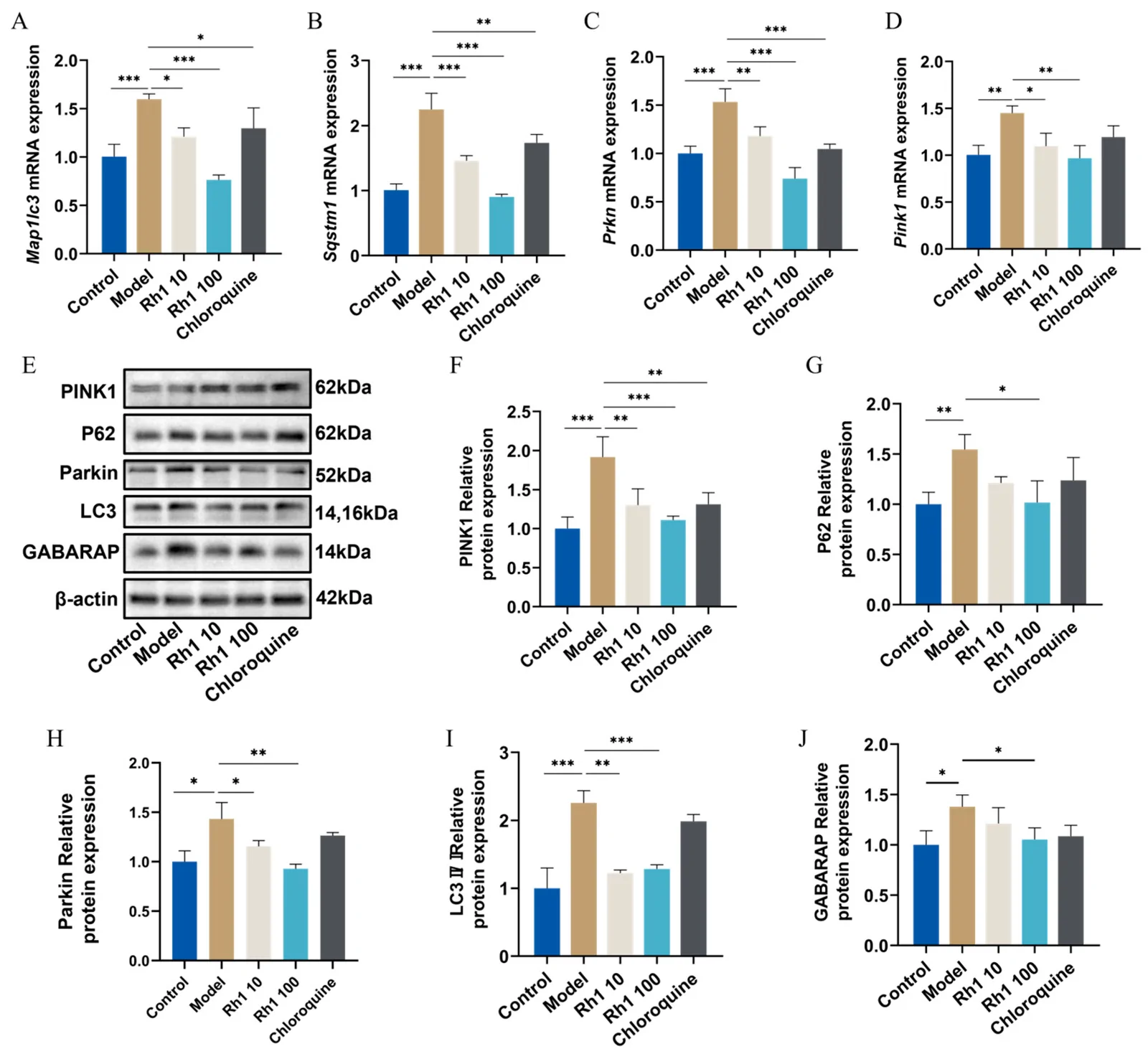
Rh1 treatment attenuated Pb-associated alterations in autophagy- and mitophagy-related markers in HT22 cells: (**A**–**D**) Relative mRNA expression levels of *Map1lc3*, *Sqstm1*, *Parkin*, and *Pink1* detected by RT-qPCR. (**E**) Representative Western blot images of P62, PINK1, Parkin, LC3, GABARAP, and β-actin. (**F**–**J**) Quantitative analysis of P62, PINK1, Parkin, LC3-II/LC3-I ratio, and GABARAP protein expression. Data are presented as the mean ± SD (*n* = 3, * *p* < 0.05, ** *p* < 0.01, *** *p* < 0.001). Control, untreated control group; Model, Pb-treated model group; Rh1 10, Pb-treated group receiving 10 μM Rh1; Rh1 100, Pb-treated group receiving 100 μM Rh1; Chloroquine, Pb-treated group receiving 10 μM chloroquine.

The regulatory effects of Rh1 on Pb-associated autophagy- and mitophagy-related signaling were further examined in HT22 cells. Chloroquine was included as a reference inhibitor of lysosome-dependent autophagic degradation. As shown in Figure 8A–D, exposure to 10 μM lead acetate trihydrate for 24 h significantly increased the mRNA expression levels of *Map1lc3*, *Sqstm1*, *Parkin*, and *Pink1* compared with the control group. Rh1 treatment reduced these Pb-associated increases, with 100 μM Rh1 showing a stronger effect than 10 μM Rh1. In the chloroquine group, the expression levels of several autophagy- and mitophagy-related genes remained relatively elevated under the present experimental conditions, although they were not consistently higher than those in the Pb model group.

Consistent with the RT-qPCR results, Western blot analysis showed that Pb exposure increased the protein expression levels of PINK1, Parkin, P62, LC3-II/LC3-I, and GABARAP in HT22 cells (Figure 8E–J). Rh1 treatment attenuated these Pb-associated alterations, particularly at 100 μM. These findings indicate that Rh1 was associated with attenuation of Pb-associated alterations in autophagy- and mitophagy-related markers in vitro, consistent with the regulatory effects observed in Pb-exposed mouse brain tissue. In the chloroquine group, several autophagy- and mitophagy-related markers remained relatively elevated compared with the Rh1-treated groups, but they did not consistently differ from the Pb model group across all measured markers.

### 2.8. Rh1 Attenuated Pb-Associated ROS Accumulation in HT22 Cells

**Figure 9:**
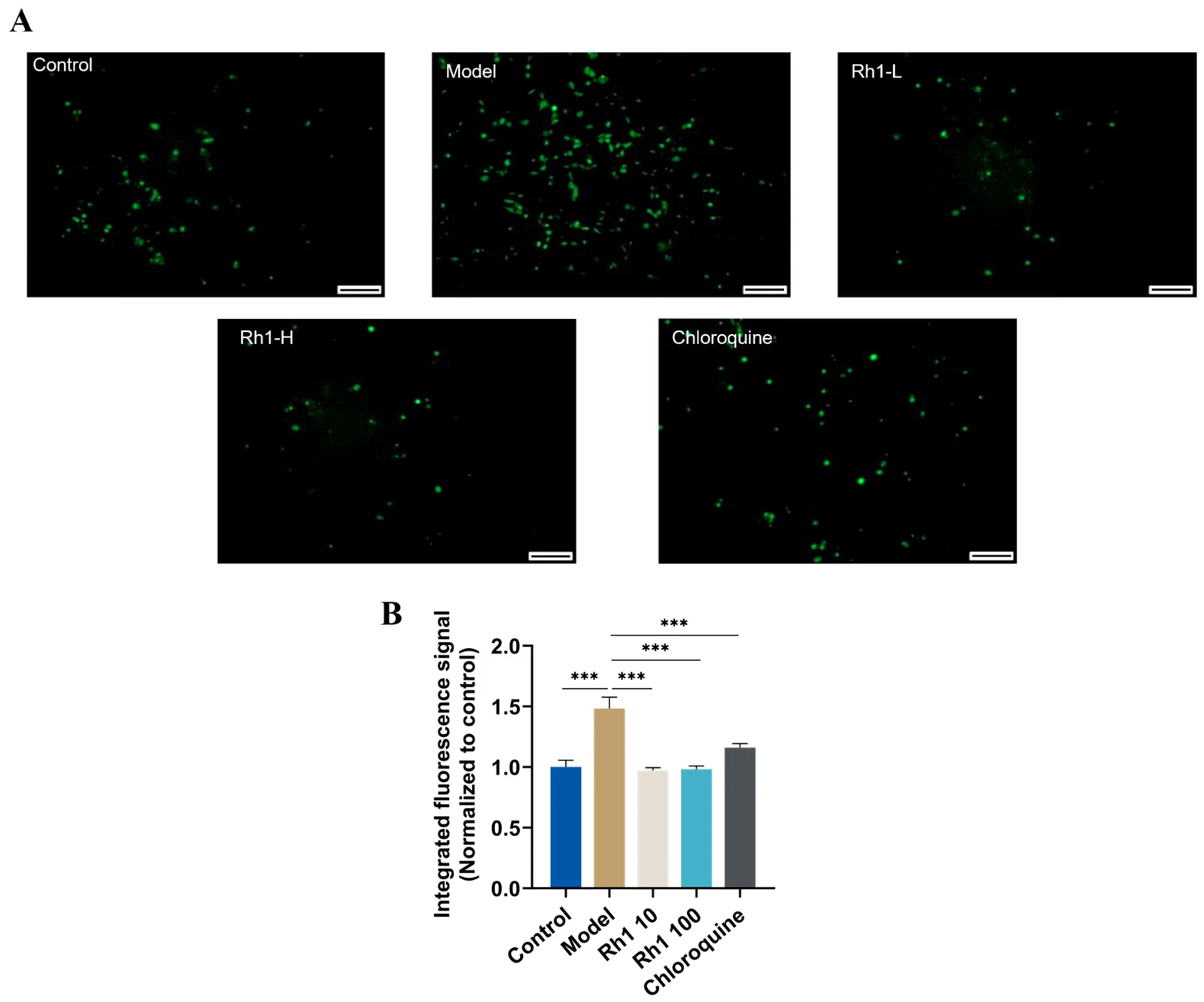
Rh1 treatment attenuated Pb-associated intracellular ROS accumulation in HT22 cells: (**A**) Representative fluorescence images of DCFH-DA staining in HT22 cells from the control, Pb model, Rh1 10, Rh1 100, and chloroquine groups (scale bar = 100 μm). (**B**) Quantitative analysis of integrated fluorescence intensity normalized to the control group. Data are presented as the mean ± SD (*n* = 3, *** *p* < 0.001). Control, untreated control group; Model, Pb-treated model group; Rh1 10, Pb-treated group receiving 10 μM ginsenoside Rh1; Rh1 100, Pb-treated group receiving 100 μM ginsenoside Rh1; Chloroquine, Pb-treated group receiving 10 μM chloroquine.

Intracellular ROS accumulation was assessed by DCFH-DA staining in HT22 cells. As shown in Figure 9A, exposure to 10 μM lead acetate trihydrate for 24 h significantly increased DCFH-DA-associated green fluorescence intensity compared with the control group, consistent with increased intracellular ROS accumulation. Treatment with 10 or 100 μM Rh1 reduced the Pb-associated fluorescence signal.

Quantitative analysis further confirmed that Pb exposure significantly increased intracellular ROS levels, whereas both Rh1 treatments significantly reduced ROS accumulation (Figure 9B). The chloroquine group maintained a relatively elevated fluorescence intensity compared with the Rh1-treated groups under the present experimental conditions. These findings indicate that Rh1 treatment was associated with attenuation of Pb-associated intracellular ROS accumulation in HT22 cells.

### 2.9. Rh1 Treatment Attenuated Pb-Associated Alterations in NKCC1/KCC2 Expression in HT22 Cells

**Figure 10:**
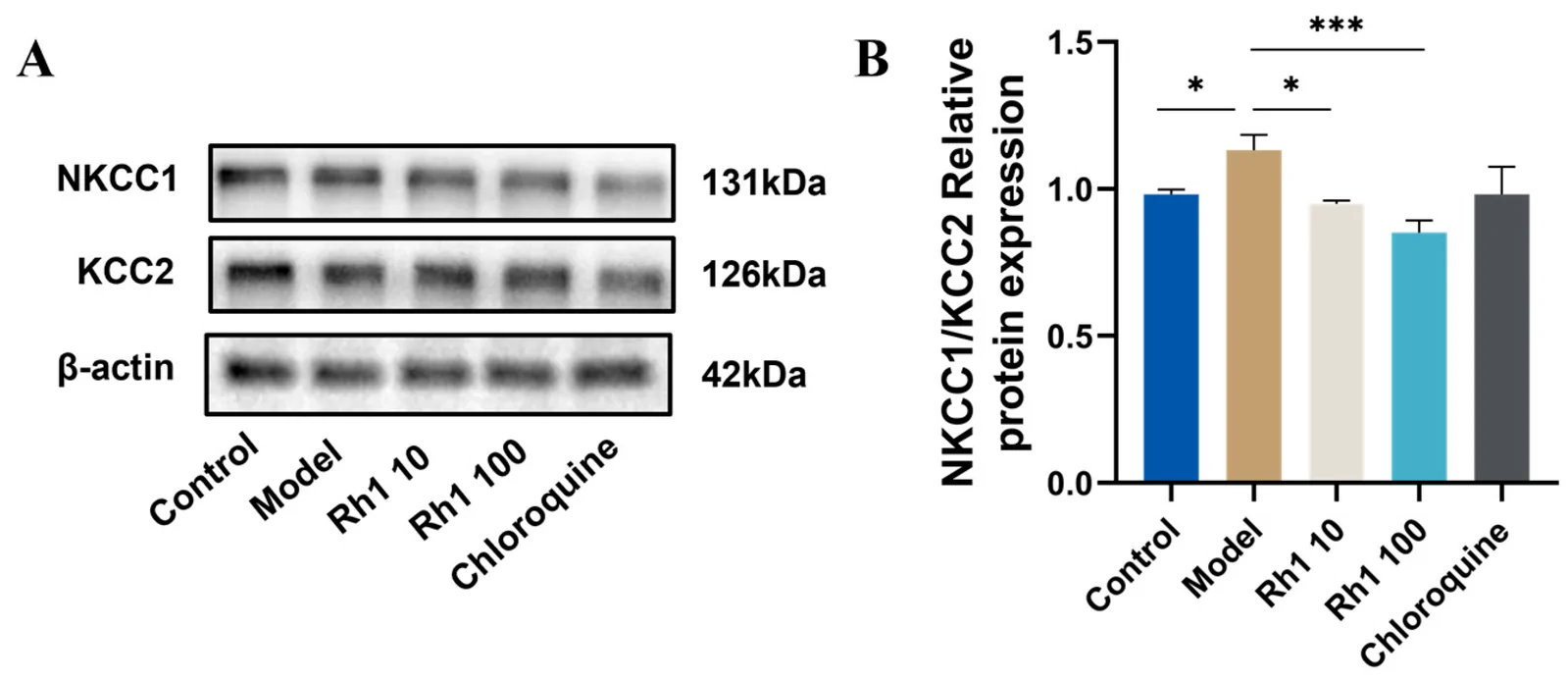
Rh1 treatment attenuated Pb-associated alterations in NKCC1/KCC2 expression in HT22 cells: (**A**) Representative Western blot images of NKCC1 and KCC2 protein expression in HT22 cells. (**B**) Quantitative analysis of the NKCC1/KCC2 protein expression ratio. Data are presented as the mean ± SD (*n* = 3, * *p* < 0.05, *** *p* < 0.001). Control, untreated control group; Model, Pb-treated model group; Rh1 10, Pb-treated group receiving 10 μM ginsenoside Rh1; Rh1 100, Pb-treated group receiving 100 μM ginsenoside Rh1; Chloroquine, Pb-treated group receiving 10 μM chloroquine.

To further examine the effect of Rh1 on chloride transporter regulation in vitro, NKCC1 and KCC2 protein expressions were assessed in HT22 cells by Western blotting. As shown in Figure 10A,B, Pb exposure significantly increased the NKCC1/KCC2 ratio compared with the control group. Treatment with Rh1 attenuated this Pb-associated increase, with 100 μM Rh1 showing a stronger regulatory effect than 10 μM Rh1. The chloroquine group maintained a relatively elevated NKCC1/KCC2 ratio under the present experimental conditions. However, the ratio was not consistently higher than that in the Pb model group. These findings indicate that Rh1 treatment was associated with attenuation of Pb-associated alterations in NKCC1/KCC2 expression in HT22 cells.

## 3. Discussion

The present study provides in vivo and in vitro evidence that ginsenoside Rh1 treatment was associated with attenuation of Pb-related neurobehavioral abnormalities, histopathological alterations, oxidative and inflammatory stress, alterations in GABA-associated regulation, and changes in mitochondrial quality-control signaling. Rather than supporting a single linear mechanism, the findings suggest that Rh1 may influence several interconnected responses to chronic Pb exposure. Importantly, the present data demonstrate parallel changes in these processes but do not establish direct causal relationships among Pb burden, mitophagy-related signaling, and GABAergic regulation.

Pb remains an important food safety and public health concern because chronic exposure may occur through contaminated food, drinking water, soil, and other environmental sources [26,27]. The behavioral and histopathological abnormalities observed in the present model are consistent with previous evidence that the hippocampus is particularly vulnerable to Pb-associated oxidative injury, synaptic dysfunction, and disturbances in learning and emotional regulation [26,27,28].

An interesting finding was the reduction in blood Pb concentrations after Rh1 supplementation and the reduction in brain Pb concentrations in the high-dose group. However, the mechanism underlying this observation remains unresolved. Rh1 may potentially influence gastrointestinal Pb absorption, fecal or urinary elimination, tissue redistribution, metal transport, intestinal barrier integrity, or blood–brain barrier function [29]. Its antioxidant and anti-inflammatory effects may also indirectly influence the function of organs and barriers involved in metal handling and excretion. Nevertheless, Pb toxicokinetics, transporter expression, excretion, and direct Rh1–Pb binding were not evaluated. Rh1 should therefore not be interpreted as a Pb-chelating compound on the basis of the current results. Future studies measuring intestinal uptake, fecal and urinary Pb excretion, time-dependent tissue distribution, blood–brain barrier transport, and direct binding capacity will be required to clarify this effect.

Inflammatory activation and oxidative stress are closely interconnected components of Pb toxicity [9,17]. Pb can disrupt endogenous antioxidant defenses, promote lipid peroxidation, impair mitochondrial function, and activate inflammatory signaling. The concurrent changes in antioxidant- and inflammation-related indicators toward control levels after Rh1 treatment are consistent with the known antioxidant and anti-inflammatory properties of ginsenosides [30,31]. These effects may create a more favorable cellular environment for preserving neuronal signaling and mitochondrial function. However, because inflammatory mediators, redox status, and mitochondrial injury can influence one another bidirectionally, the present data do not identify whether suppression of oxidative stress preceded the other molecular changes or occurred as part of a broader protective response.

The changes in GABA content and NKCC1/KCC2 expression are potentially relevant to Pb-associated disturbances in neuronal inhibition. KCC2-mediated chloride extrusion is important for maintaining low intracellular chloride concentrations and hyperpolarizing GABA_A_ receptor signaling in mature neurons, whereas increased chloride-loading capacity and/or reduced relative KCC2 function may weaken inhibitory efficacy [13,14]. Therefore, the Pb-associated increase in the NKCC1/KCC2 ratio observed in the present study could be consistent with a shift in excitation–inhibition balance toward greater neuronal excitability. Such a shift may contribute to altered hippocampal network activity and the behavioral abnormalities observed after Pb exposure. However, this interpretation requires caution because NKCC1 is not restricted to neurons in the adult brain. Kurki et al. reported that NKCC1 protein is prominently expressed in oligodendrocytes and is also detectable in astrocytes, microglia, and dentate gyrus progenitor cells, whereas its expression in mature neuronal somata is relatively low [32]. Accordingly, because the present Western blot analyses were performed using whole-brain tissue homogenates, the measured NKCC1/KCC2 ratio cannot be interpreted as a neuron-specific indicator of intracellular chloride regulation or GABAergic function. The altered ratio may therefore reflect a combination of changes in neuronal KCC2 expression and NKCC1 expression in neuronal and non-neuronal cell populations. In oligodendrocytes, astrocytes, and microglia, NKCC1 may contribute to ionic and water homeostasis, myelin-associated function, regulation of the extracellular environment, and inflammatory or structural responses to injury [32]. Thus, Pb-associated alterations in NKCC1/KCC2 expression may have broader implications for neuron–glia interactions, extracellular ionic homeostasis, myelin function, and network excitability, rather than reflecting only a shift in neuronal GABA polarity. Attenuation of the Pb-associated increase in the NKCC1/KCC2 ratio, together with the increase in brain GABA content, is therefore compatible with changes in GABA-associated regulation and in the cellular environment relevant to excitation–inhibition balance. Nevertheless, these measurements do not demonstrate complete restoration of GABAergic homeostasis or functional recovery of GABAergic neurotransmission. Cell-type-specific localization studies, intracellular chloride measurements, and electrophysiological assessment of inhibitory postsynaptic currents and GABA_A_ receptor-mediated responses will be required to determine the relative contributions of neuronal and glial chloride-transporter changes to the present findings.

Mitochondrial dysfunction may provide another biological context in which Pb exposure is associated with neuronal signaling abnormalities. Neuronal inhibition and neurotransmitter metabolism depend on mitochondrial ATP production and redox balance, while GABA metabolism is connected to the tricarboxylic acid cycle through the GABA shunt [33,34]. The observed changes in PINK1, Parkin, LC3-II/LC3-I, P62, and GABARAP indicate alterations in autophagy- and mitophagy-related signaling under Pb exposure. However, accumulation of LC3-II and P62 can reflect either increased autophagosome formation or impaired lysosomal degradation. These markers therefore cannot, by themselves, establish enhanced or restored mitophagic flux.

Chloroquine was included as a reference inhibitor of lysosome-dependent autophagic degradation. Under Pb exposure, the chloroquine group maintained relatively elevated levels of several autophagy-related markers, ROS, and the NKCC1/KCC2 ratio, although these indicators did not consistently differ from the Pb model group. These observations are compatible with the possibility that impaired autophagic clearance may be associated with Pb-associated cellular stress, but they do not provide direct evidence of mitophagic flux or prove that Rh1 acts through autophagy regulation. Tandem fluorescent LC3 reporters, mitochondria-specific mitophagy reporters, lysosomal functional assays, and targeted genetic manipulation of PINK1/Parkin-related pathways will be needed to clarify these processes.

The concurrent changes in GABA-associated markers and mitochondrial quality-control signaling raise the possibility of an interaction between neuronal inhibition and mitochondrial stress. Mitochondrial dysfunction could reduce metabolic support for GABA synthesis and turnover, whereas weakened inhibition could increase neuronal excitability and energy demand. Rh1 treatment may influence these potentially interconnected processes through changes in Pb burden, redox status, inflammation, and mitochondrial signaling. However, the present data do not establish that modulation of mitophagy directly causes the observed changes in GABA-associated regulation. Functional mitophagic flux assays and pathway-specific interventions will be required to determine whether mitochondrial quality-control changes contribute causally to altered GABAergic function.

From a translational perspective, Rh1 should currently be considered a ginseng-derived bioactive compound of experimental interest whose practical application remains to be established. The present findings were obtained in a mouse model and an HT22 cell model, and the bioavailability, pharmacokinetics, biologically effective dose range, long-term safety, and efficacy of Rh1 in humans remain unknown.

Although the present study specifically investigated Pb-associated neuronal injury, oxidative stress, inflammatory activation, mitochondrial dysfunction, and disruption of cellular homeostasis are also involved in the toxicity of other heavy metals, including Cd and Cr [35,36]. These partially shared pathological features raise the possibility that the antioxidant, anti-inflammatory, and mitochondrial regulatory properties of Rh1 may also be relevant in other metal-exposure contexts. Previous studies have reported that certain ginseng-derived compounds attenuate Cd- or Cr-associated toxic responses, primarily in association with changes in oxidative stress and inflammatory signaling [36,37]. However, evidence regarding Rh1 itself remains limited, and the toxicokinetics, target organs, molecular pathways, and severity of injury differ among metals according to chemical form, dose, route and duration of exposure, and host susceptibility. Therefore, the present findings should not be generalized beyond Pb exposure. Whether Rh1 can protect against Cd-, Cr-, or other heavy metal-associated toxicity requires direct evaluation using metal-specific cellular and animal models.

In addition, the relatively small sample size used for several biochemical and molecular analyses should be considered when interpreting these findings. Future studies with larger, prospectively determined sample sizes, direct functional assays, and ultimately appropriately designed human studies will be necessary to evaluate the reproducibility, mechanistic basis, safety, and translational relevance of Rh1.

## 4. Materials and Methods

### 4.1. Chemicals and Reagents

Lead acetate trihydrate (Cat# 316512, Sigma-Aldrich, Saint Louis, MO, USA) was used to establish the Pb exposure model. Ginsenoside Rh1, a ginseng-derived bioactive compound with a purity of ≥98%, was obtained from Biopurify Phytochemicals Ltd. (Cat# BP0667, Chengdu, China). High-glucose Dulbecco’s modified Eagle’s medium (DMEM), fetal bovine serum (FBS), and penicillin–streptomycin solution were purchased from Gibco (Thermo Fisher Scientific, Waltham, MA, USA). CCK-8 and Reactive Oxygen Species Assay Kit based on DCFH-DA staining were obtained from Beyotime Biotechnology (Shanghai, China). TRIzol reagent, cDNA Synthesis SuperMix, and SYBR Green Master Mix were purchased from Yeasen Biotechnology (Shanghai, China). RIPA lysis buffer, protease/phosphatase inhibitor cocktail, and bicinchoninic acid (BCA) protein assay kit were obtained from Thermo Fisher Scientific (Waltham, MA, USA).

Primary antibodies against NKCC1, KCC2, PINK1, Parkin, LC3, P62/SQSTM1, GABARAP, and β-actin were purchased from Proteintech (Wuhan, China). The GABA content assay kit was obtained from Solarbio Life Sciences (Beijing, China). Commercial enzyme-linked immunosorbent assay (ELISA) kits for IL-1β, TNF-α, and IL-6 were obtained from Jianglai Bioengineering Co., Ltd. (Shanghai, China). Assay kits for MDA, GSH, and SOD activity were purchased from Nanjing Jiancheng Bioengineering Institute (Nanjing, China). Ultra-pure concentrated nitric acid (65%, *v*/*v*) was used for sample digestion. All other chemicals and reagents were of analytical grade unless otherwise stated.

### 4.2. Animals and Experimental Design

Male C57BL/6J mice, aged 3–4 weeks and weighing 20–25 g, were obtained from Sibeifu (Beijing, China) Biotechnology Co., Ltd. Mice were housed under standard laboratory conditions at 22 ± 2 °C with a 12 h light/dark cycle and had free access to standard chow and water. A separate publicly registered study protocol was not prepared. The research question, key experimental design features, and planned outcome assessments were described in the animal ethics application, which was reviewed and approved before the start of the study by the Institutional Animal Care and Use Committee of the College of Basic Medical Science of Jilin University (approval No. 2025-679).

After a 7-day acclimatization period, all mice were examined to confirm their general health status, and animals with obvious abnormalities were excluded. Thirty-two healthy mice were then randomly assigned to four groups: control, Pb model, Pb + Rh1 low-dose group, and Pb + Rh1 high-dose group, with eight mice in each group. The sample size was determined based on previous comparable animal studies investigating Pb-induced toxicity and pharmacological intervention models [38,39]. No additional inclusion or exclusion criteria for animals or experimental units were established a priori, and no animals, experimental units, or data points were excluded during the experiment or subsequent analysis.

The low and high doses of Rh1 were set at 5 and 20 mg/kg body weight, respectively, based on previously published studies of ginsenosides and related natural bioactive compounds [40,41]. Animals from different groups were maintained under the same environmental conditions, and treatments and outcome measurements were performed using the same procedures across groups to minimize potential confounders. Group allocation was performed by an investigator who was not involved in outcome assessment or data analysis. Investigators responsible for animal treatment were aware of group allocation because of the different experimental interventions. Outcome assessment and data analysis were performed using coded samples by investigators blinded to group allocation. Group identities were revealed only after completion of the analysis.

To reduce pain and suffering, all procedures were performed by trained personnel, and animals were handled gently to minimize stress. Animals were monitored daily for general appearance, body weight, food and water intake, posture, fur condition, and signs of pain. Animals showing a loss of ≥15% of baseline body weight were subjected to intensified monitoring, and a loss of ≥20% of baseline body weight was considered a humane endpoint requiring euthanasia. Additional humane endpoint criteria included persistent inability to access food or water, severe lethargy or impaired mobility, labored breathing, or other signs of substantial distress.

### 4.3. Pb Exposure and Rh1 Supplementation

Chronic Pb exposure was established by providing mice with drinking water containing lead acetate at a final concentration of 100 ppm from week 1 to week 10. This oral exposure protocol was used to model sustained Pb intake relevant to environmental and food safety concerns. Rh1, a ginseng-derived bioactive compound, was used as the dietary functional factor in the intervention phase. Rh1 supplementation was initiated at week 7 and continued until the end of week 10. Rh1 was dissolved in corn oil and administered once daily by intragastric gavage at doses of 5 or 20 mg/kg body weight, with an administration volume of 10 μL/g body weight. Mice in the control and Pb model groups received an equal volume of corn oil vehicle using the same gavage procedure. Body weight and general health status were monitored throughout the experimental period.

### 4.4. Behavioral Tests

Behavioral assessments were performed during the 10th week of Pb exposure, before the end of the experimental period, in a quiet and dimly lit room. Mice were allowed to acclimatize to the testing room for 12 h before behavioral testing. To minimize olfactory cues, the apparatus was cleaned with 75% ethanol between trials.

#### 4.4.1. Y-Maze Test

Spatial recognition and exploratory behavior were evaluated using a two-trial novel-arm recognition paradigm in a Y-maze apparatus. During the 5 min training trial, one arm was blocked as the novel arm, and mice were allowed to explore the two accessible arms. After a 1 h intertrial interval, the novel arm was opened, and mice were allowed to explore all three arms for 5 min. The number of entries into the novel arm and the distance traveled in the novel arm were recorded using an automated video-tracking system (Smart 3.0, Panlab, Cornellà de Llobregat, Spain).

#### 4.4.2. Elevated Plus Maze

Anxiety-like behavior was assessed using the elevated plus maze, which consisted of two open arms and two closed arms elevated 50 cm above the floor. Each mouse was placed on the central platform facing an open arm and allowed to explore freely for 5 min. The distance traveled and percentage of time spent in the open arms were recorded as indicators of exploratory activity and anxiety-like behavior.

#### 4.4.3. Open-Field Test

The open-field test was performed in a square arena (50 × 50 × 40 cm) to assess spontaneous locomotor activity and anxiety-like behavior. Each mouse was placed in the center of the arena and allowed to explore freely for 5 min. The time spent in the central zone and total locomotor activity were recorded.

### 4.5. Histopathological Analysis

After behavioral testing, mice were anesthetized, and brain tissues were collected and fixed in 4% paraformaldehyde for 48 h. Samples were dehydrated through a graded ethanol series, embedded in paraffin, and sectioned at 5 μm thickness. Sections were deparaffinized, rehydrated, and stained with H&E. Histopathological changes, including neuronal arrangement, pyknosis, and cell loss, were examined under a light microscope (Olympus, Tokyo, Japan).

### 4.6. Determination of Pb Levels, Inflammatory Cytokines, and Oxidative Stress Biomarkers

After completion of behavioral testing, mice were anesthetized, and blood and brain tissue samples were collected for biochemical analyses. For Pb determination, whole blood samples were collected by orbital exsanguination into heparinized centrifuge tubes. For serum cytokine analysis, blood samples were collected into non-anticoagulant tubes, allowed to clot at room temperature, and centrifuged to obtain serum. Brain tissue samples were rapidly isolated, rinsed with ice-cold saline, and stored appropriately until analysis.

For elemental analysis, whole blood aliquots and brain tissue samples were digested in 10 mL of ultra-pure concentrated nitric acid (65%, *v*/*v*) using a closed-vessel microwave digestion system with the following thermal program: 2 h at 100 °C, 2 h at 120 °C, 3 h at 140 °C, and 1 h at 180 °C. The digested samples were evaporated to near dryness, approximately 0.1 mL, on a graphite hot plate at 110–150 °C, allowed to cool, and then diluted to 10 mL with Milli-Q water. Pb quantification was performed using a NEXION 300X inductively coupled plasma mass spectrometer (PerkinElmer, Waltham, MA, USA). Procedural blanks and standard reference materials were included in all analytical runs for quality control.

Serum levels of IL-1β, TNF-α, and IL-6 were determined using commercial ELISA kits according to the manufacturer’s instructions. Absorbance was measured using a microplate reader, and cytokine concentrations were calculated according to the standard curves.

Oxidative stress-related biomarkers in brain tissue were assessed by measuring GSH content, MDA level, and SOD activity using commercial assay kits according to the manufacturer’s protocols. Brain tissues were homogenized in ice-cold extraction buffer and centrifuged at 4 °C. The supernatants were collected for subsequent biochemical assays.

### 4.7. Cell Culture and Treatment

HT22 cells were cultured in high-glucose DMEM supplemented with 10% FBS and 1% penicillin–streptomycin at 37 °C in a humidified atmosphere containing 5% CO_2_. For experiments, cells were seeded and allowed to adhere overnight. Pb-induced cellular injury was established by exposing cells to 10 μM lead acetate for 24 h. Rh1 was administered at concentrations of 10 and 100 μM. Chloroquine was included as a reference inhibitor of lysosome-dependent autophagic degradation. In the chloroquine group, cells were simultaneously treated with 10 μM lead acetate and 10 μM chloroquine for 24 h. Cells were divided into five groups: control, model, Rh1 10, Rh1 100, and chloroquine.

### 4.8. CCK-8 Assay

Cell viability was determined using the CCK-8 assay. HT22 cells were seeded in 96-well plates at a density of 5 × 10^3^ cells/well and treated with Pb and/or Rh1 for 24 h. Subsequently, 10 μL of CCK-8 solution was added to each well, followed by incubation at 37 °C for 40 min. Absorbance was measured at 450 nm using a microplate reader (Tecan Group Ltd., Grödig, Austria).

### 4.9. ROS Staining

Intracellular ROS production was detected using DCFH-DA staining. After treatment, HT22 cells were incubated with 10 μM DCFH-DA in serum-free medium for 20 min at 37 °C in the dark. Cells were then washed three times with PBS to remove excess probe. Fluorescence images were captured using a fluorescence microscope (Olympus BX53, Tokyo, Japan) under identical exposure settings. Integrated fluorescence intensity was quantified using ImageJ software (version1.6.0, NIH, Bethesda, MD, USA) and normalized to the control group.

### 4.10. Western Blot Analysis

Brain tissues or HT22 cells were lysed in RIPA buffer containing protease and phosphatase inhibitors. Protein concentrations were determined using a BCA protein assay kit. Equal amounts of protein were separated by SDS-PAGE and transferred onto PVDF membranes. After blocking with 5% non-fat milk for 2 h at room temperature, membranes were incubated with primary antibodies overnight at 4 °C. Membranes were then washed and incubated with HRP-conjugated secondary antibodies for 2 h at room temperature. Protein bands were visualized using ECL reagent and quantified using ImageJ software. β-actin was used as the internal loading control.

### 4.11. RT-qPCR

Total RNA was extracted using TRIzol reagent according to the manufacturer’s instructions. RNA concentration was determined using a NanoDrop One spectrophotometer. cDNA was synthesized from 1 μg of total RNA. Quantitative PCR was performed using SYBR Green Master Mix on a real-time PCR system (Bio-Rad, Hercules, CA, USA). Relative mRNA expression was calculated using the 2^−ΔΔCt^ method and normalized to β-actin. Primer sequences, amplification efficiencies, slopes, and coefficients of determination (R^2^) are provided in Table S1, and representative melting curve analyses are shown in Figure S1.

### 4.12. GABA Measurement

GABA content in brain tissues was measured using a commercial GABA assay kit. Brain tissues were homogenized in extraction buffer on ice and centrifuged at 4 °C. The supernatants were collected and processed according to the manufacturer’s protocol. Absorbance was measured at 640 nm, and results were expressed as μmol/g tissue.

### 4.13. Statistical Analysis

Data are presented as the mean ± standard deviation (SD). Statistical analyses were performed using GraphPad Prism 9.0 (GraphPad Software, Boston, MA, USA). Differences among multiple groups were analyzed by one-way analysis of variance (ANOVA) followed by Tukey’s post hoc test. A value of *p* < 0.05 was considered statistically significant.

## 5. Conclusions

In conclusion, this study provides in vivo and in vitro evidence that Rh1, a ginseng-derived bioactive compound, was associated with attenuation of chronic Pb-associated neuronal injury, reductions in Pb burden and oxidative stress, attenuation of Pb-related alterations in GABA content and NKCC1/KCC2 expression, and modulation of mitophagy-related signaling. These results support further investigation of Rh1 as a ginseng-derived bioactive compound with potential protective relevance to Pb-associated neurotoxicity.

## Data Availability

Data will be made available upon request.

## Supplementary Materials

The following supporting information can be downloaded at https://www.mdpi.com/article/10.3390/molecules31162801/s1, Figure S1: Melting curve analysis of qRT-PCR products. Melting curves of three representative samples are shown for each primer pair targeting (A) *Sqstm1*, (B) *Parkin*, (C) *Pink1*, (D) *Map1lc3b*, and (E) *β-Actin*. A single predominant melting peak was observed for each primer pair, supporting the specificity of the amplification products; Table S1: Primer sequences used for qRT-PCR amplification of mouse genes.

## Abbreviations

The following abbreviations are used in this manuscript:

GABA	Gamma-aminobutyric acid
NKCC1	Sodium–potassium–chloride cotransporter 1
KCC2	Potassium–chloride cotransporter 2
GSH	Glutathione
SOD	Superoxide dismutase
SD	Standard deviation
H&E	Hematoxylin and eosin
IL-1β	Interleukin-1β
TNF-α	Tumor necrosis factor-α
IL-6	Interleukin-6
MDA	Malondialdehyde
ELISA	Enzyme-linked immunosorbent assay
CCK8	Cell counting kit-8
