# Supplementary material for: Ginsenoside Rh1 Modulates GABAergic Inhibitory Homeostasis and Mitochondrial Quality Control During Lead (Pb)-Associated Neuronal Dysfunction
Source: Molecules. 2026 Aug 11;31(16):2801. doi: 10.3390/molecules31162801 (PMC13515940; doi:10.3390/molecules31162801)
Supplement: Supplementary file 1 [file molecules-31-02801-s001.zip › molecules-4441173-supplementary.pdf]

Table S1. Primer sequences used for qRT-PCR amplification of mouse genes.

Gene	Forward primer	Reverse primer	Amplification efficiency（%）	Slope	R^2
Sqstm1	GAACTCGCTATAAGTGCAGTGT	AGAGAAGCTATCAGAGAGGTGG	93.425	-3.49	0.999
Parkin	TCTTCCAGTGTAACCACCGTC	GGCAGGGAGTAGCCAAGTT	100.060	-3.32	0.999
Pink1	TTCTTCCGCCAGTCGGTAG	CTGCTTCTCCTCGATCAGCC	97.078	-3.39	0.996
Map1lc3b	TTATAGAGCGATACAAGGGGGAG	CGCCGTCTGATTATCTTGATGAG	97.264	-3.34	0.997
β-Actin	CATTGCTGACAGGATGCAGAAGG	TGCTGGAAGGTGGACAGTGAGG	95.657	-3.42	0.999

Supplementary Figure S1

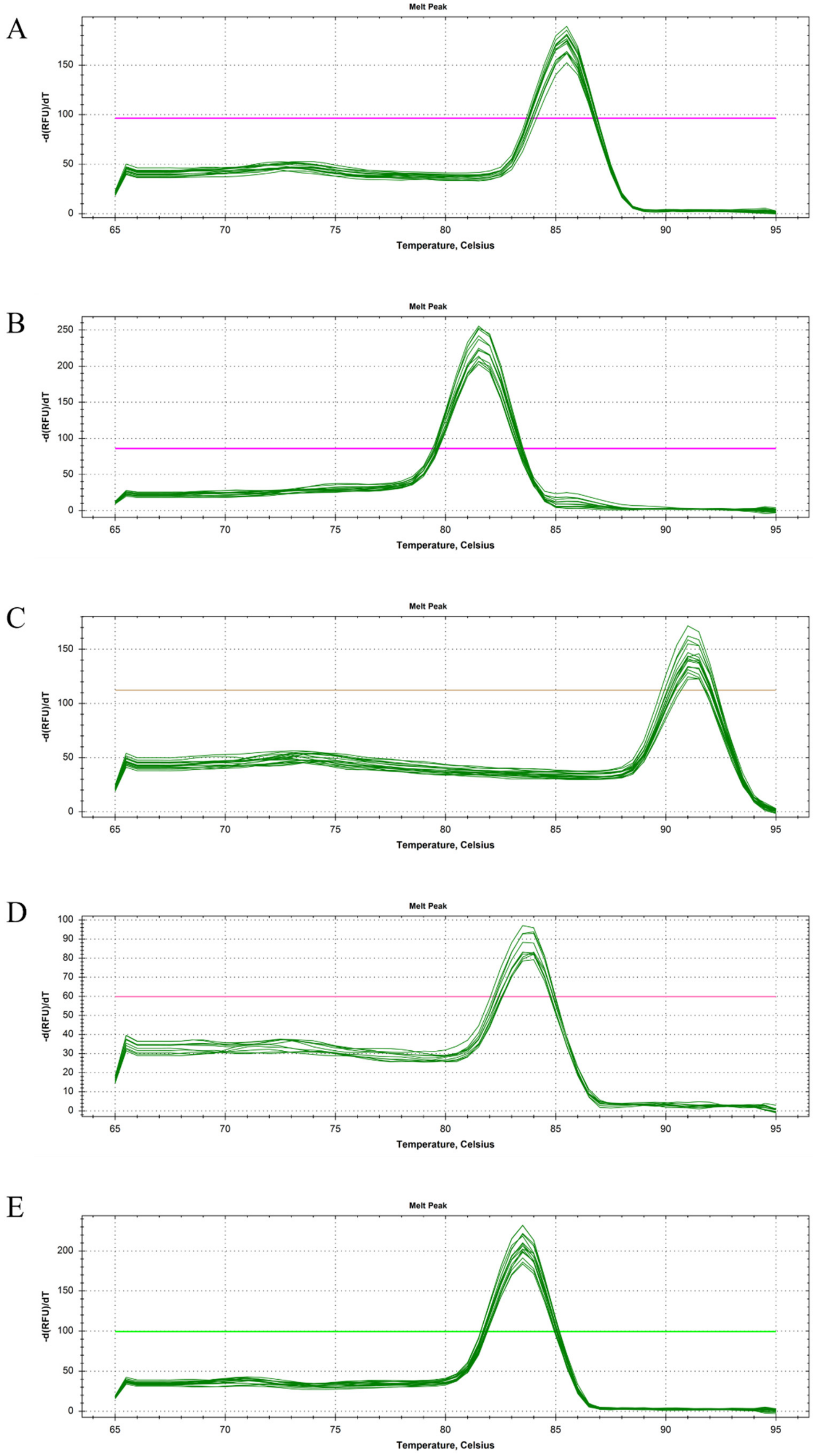

Figure S1. Melting curve analysis of qRT-PCR products. Melting curves of three representative samples are shown for each primer pair targeting (A) *Sqstm1*, (B) *Parkin*, (C) *Pink1*, (D) *Map1lc3b*, and (E) *β-Actin*. A single predominant melting peak was observed for each primer pair, supporting the specificity of the amplification products.
